# Surveillance of hemodialysis related infections: a prospective multicenter study

**DOI:** 10.1038/s41598-022-24820-3

**Published:** 2022-12-23

**Authors:** Imran Hasanoglu, Rahmet Guner, Suzan Sahin, Fatma Yılmaz Karadag, Ergun Parmaksiz, H. Veli Atalay, Sabahat Alısır Ecder, Tugba Arslan Gulen, Zuhal Atan Ucar, Oguz Karabay, Savas Sipahi, Esra Kaya Kılıc, Murat Duranay, Derya Yapar, İbrahim Dogan, Gulden Ersoz, Gulcan Turkmen, Ahmet Alper Kıykım

**Affiliations:** 1 Department of Infectious Diseases and Clinical Microbiology, Ankara City Hospital, Yildirim Beyazit University School of Medicine, Bilkent, Ankara, Turkey; 2 Department of Infectious Diseases and Clinical Microbiology, Dr. Lütfi Kırdar Kartal Education and Research Hospital, Istanbul, Turkey; 3 Department of Infectious Diseases and Clinical Microbiology, Health Sciences University, Sancaktepe Sehit Professor Doctor Ilhan Varank Education and Research Hospital, Istanbul, Turkey; 4 Department of Nephrology, Dr. Lütfi Kırdar Kartal Education and Research Hospital, Istanbul, Turkey; 5 Department of Nephrology, Gulhane Education and Research Hospital, Ankara, Turkey; 6 Department of Nephrology, Prof. Dr. Suleyman Yalcın City Hospital Medeniyet University Faculty of Medicine, Istanbul, Turkey; 7 Department of Infectious Diseases and Clinical Microbiology, University of Health Sciences Adana City Hospital, Adana, Turkey; 8 Department of Nephrology, Liv Hospital Vadistanbul, Istanbul, Turkey; 9 Department of Infectious Diseases and Clinical Microbiology, Sakarya University, Sakarya, Turkey; 10 Department of Nephrology, Sakarya University, Sakarya, Turkey; 11 Department of Infectious Diseases and Clinical Microbiology, Ankara Education and Research Hospital, Ankara, Turkey; 12 Department of Nephrology, Ankara Education and Research Hospital, Ankara, Turkey; 13 Department of Infectious Diseases and Clinical Microbiology, Hitit University, Çorum, Turkey; 14 Department of Nephrology, Hitit University, Çorum, Turkey; 15 Department of Infectious Diseases and Clinical Microbiology, Mersin University Hospital, Mersin, Turkey; 16 Department of Nephrology, Mersin University Hospital, Mersin, Turkey; 17 Infection Control Committee, Mersin University Hospital, Mersin, Turkey

**Keywords:** Infectious diseases, Haemodialysis

## Abstract

As in many countries, there is neither a surveillance system nor a study to reveal the hemodialysis (HD) related infection rates in Turkey. We aimed to investigate the infection rate among HD outpatients and implement CDC’s surveillance system. A multicenter prospective surveillance study is performed to investigate the infection rate among HD patients. CDC National Healthcare Safety Network (NHSN) dialysis event (DE) protocol is adopted for definitions and reporting. During April 2016–April 2018, 9 centers reported data. A total of 199 DEs reported in 10,035 patient-months, and the overall DE rate was 1.98 per 100 patient-months. Risk of blood culture positivity is found to be 17.6 times higher when hemodialysis was through a tunneled catheter than through an arteriovenous fistula. DE rate was significantly lower in patients educated about the care of their vascular access site. *Staphylococcus aureus* was the most causative microorganism among mortal patients. Outcomes of DEs were hospitalization (73%), loss of vascular access (18.2%), and death (7.7%). This first surveillance study revealed the baseline status of HD related infections in Turkey and showed that CDC National Healthcare Safety Network (NHSN) DE surveillance system can be easily implemented even in a high workload dialysis unit and be adopted as a nationwide DE surveillance program.

## Introduction

With the prolongation of the average life span, the frequency of chronic diseases in all over the world is increasing. In Turkey, while the point prevalence of end-stage chronic renal failure (ESCRF) requiring renal replacement therapy (RRT) was 314 per million population in 2001, this number tripled and reached 988.4 per million population by the end of 2018. After cardiovascular/ cerebrovascular diseases and malignancies, infections are the fourth most common cause of death in hemodialysis (HD) patients^1^.

Monitoring of HD related infections in dialysis patients constitutes an important part of prevention. American Center for Disease Prevention and Control (CDC) implemented a surveillance program reporting dialysis event (DE) regarding infection related adverse events of HD in 1999. This surveillance program initially gathered data from voluntarily participating HD centers. Afterwards, the program progressed into a mandatory system all HD units registered. However, as in many countries, there is neither a surveillance system nor a study to reveal the HD related infection rates in Turkey. Therefore, it is crucial to emphasize the importance of the subject, to encourage monitoring this group of patients, and make data available for comparison. Benchmarks are very important in infection control. It is essential to access data that has been collected and presented in a uniform manner. This is important for both setting a standard for improvement studies and standardizing surveillance. In this study, we aimed to investigate the infection rate among HD outpatients, reveal the characteristics and risk factors of the patients, and implement CDC’s surveillance system.

## Results

During April 2016–April 2018, 9 centers from 7 different provinces reported at least 1 month of DE data to the system. Nine hundred forty-nine patients were included in the study. Characteristics of the patients are given in Table 1. Among all the patients, 741 (78.1%) had at least one co-morbidity.

**Table 1:** Characteristics of the patients (n = 949).

**Demographics**	
Age (years), mean (range)	59 (9–89)
Male, n (%)	536 (56.4)
**Years of HD, mean (range)**	6.7 (0–32)
**Addiction, n (%)**	
Cigarette	129 (13.59)
Alcohol	15 (1.58)
Drug	2 (0.21)
**Comorbidity, n (%)**	
Hypertension	556 (58.6)
Diabetes mellitus	290 (30.6)
Coronary arterial disease	148 (15.6)
Asthma / Chronic obstructive pulmonary disease	60 (6.3)
Cerebrovascular disease	22 (2.3)
At least one co-morbidity	741 (78.1)
**Immunization status, n (%)**	
Influenza vaccine	306 (32.2)
Pneumococcal vaccine	55 (5.8)
**Received training for vascular access care, n (%)**	
Yes	764 (80.5)
No	185 (19.5)
**Type of vascular access, n (%)**	
Tunneled catheter	192 (19.01)
Non-tunneled catheter	234 (23.17)
AV fistula	577 (57.13)
AV graft	7 (0.69)
**Type of hospital where the catheter inserted, n (%)**	
Education and Research Hospital	634 (62.8)
University Hospital	160 (15.8)
State Hospital	117 (11.6)
Private Hospital	99 (9.8)

**Figure 1:**
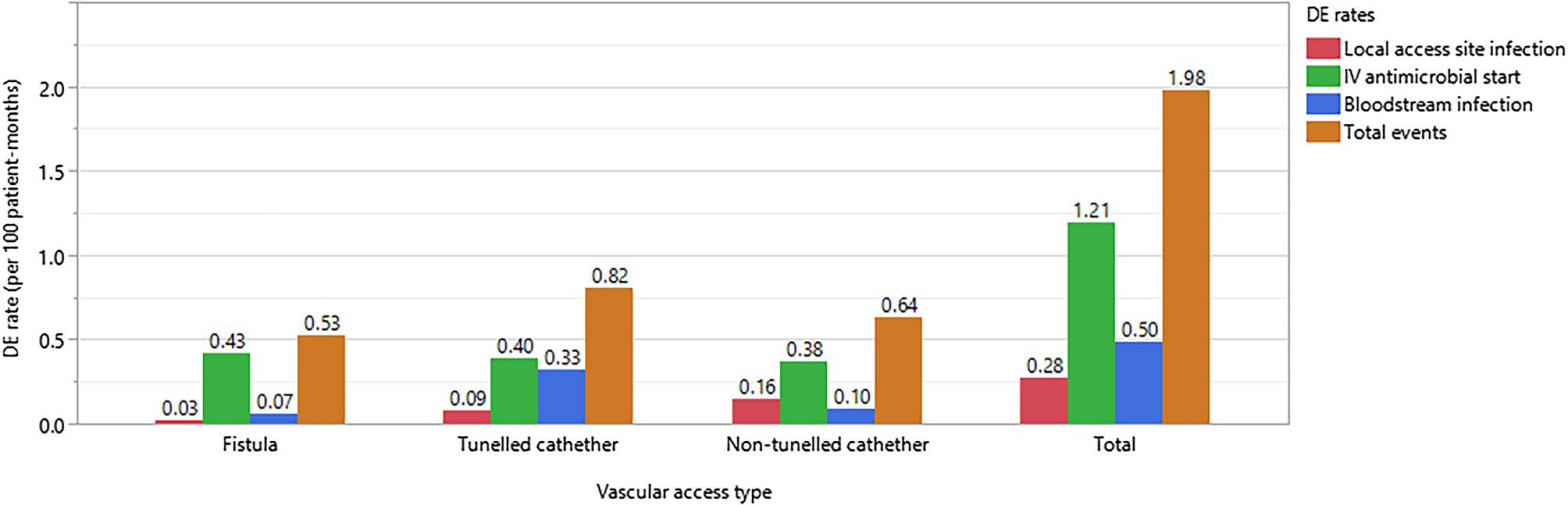
DE rates for each vascular access type.

A total of 199 DEs reported in 10,035 patient-months, and the overall DE rate was 1.98 per 100 patient-months during the surveillance period. Data for each event type and vascular access type are given in Fig. 1. Eighty-point five percent of the patients had received training for vascular access care. DE rate was significantly lower in patients educated about the care of their vascular access site (*p* < 0.0001). Mean body mass index (BMI) was significantly higher in patients with any DE (*p*:0.009).

DEs were significantly more common in patients with catheters (tunneled or non-tunneled) compared to patients with AV fistulas (*p* < 0.0001). The risk of blood culture positivity is found to be 17.6 times higher (%95 CI 7.21–43.08) when hemodialysis was through a tunneled catheter than through an arteriovenous fistula. Sixty-two-point eight percent of the catheters were inserted in a teaching and research hospital. We found no relationship between DE and the type of hospital where central venous catheter (CVC) was inserted. Outcomes of DEs were hospitalization (73%), loss of vascular access (18.2%), and death (7.7%).

Isolated microorganisms from blood cultures are given in Table 2. Fifty-four percent of the microorganisms were Gram positive. Methicillin resistance was 1% in staphylococcus. Third generation cephalosporin resistance was 50% among *Enterobacteriaceae*. *Staphylococcus aureus* was the most causative microorganism among mortal patients.

**Table 2:** Isolated microorganisms from blood cultures.

Microorganism	Fistula	Catheter	Total, n (%)
*Staphylococcus coagulase-negative*	3	7	10 (27)
*Staphylococcus aureus*	2	5	7 (18.9)
*Escherichia coli*	1	5	6 (16.2)
*Klebsiella pneumonia*	1	3	4 (10.8)
*Enterobacter cloaca*	1	2	3 (8.1)
*Pseudomonas aeruginosa*	0	3	3 (8.1)
*Enterococcus faecalis*	0	2	2 (5.4)
*Candida spp*	0	1	1 (2.7)
*Enterococcus faecium*	1	0	1 (2.7)
Total	9	28	37 (100)

## Discussion

In this multicenter prospective surveillance study, a total of 199 DEs reported in 10,035 patient-months, and the overall DE rate was 1.98 per 100 patient-months. Outcomes of DEs were hospitalization (73%), loss of vascular access (18.2%), and death (7.7%). This first surveillance study revealed the baseline status of HD related infections in Turkey and showed that CDC National Healthcare Safety Network (NHSN) DE surveillance system can be easily implemented even in a high workload dialysis unit and be adopted as a nationwide DE surveillance program.

Despite the advances in dialysis technology, infections that develop in hemodialysis patients remain important, and prevention of these infections is mainly based on surveillance. Data from the National Nephrology, Dialysis and Transplantation Registry Report of Turkey showed that with a rate of 9.98%, infections are one of the most common death causes in HD patients. Type of vascular access is important for the risk of infection. Now, it is a well-known fact that, AV fistula has the lowest risk of infection. Therefore, it has been a core aim to increase its usage as a prevention measure for dialysis events. “Fistula First” initiative of the Centers for Medicare and Medicaid Services campaigned for use of AV fistula and set objectives to increase AV fistula usage above 66% meanwhile decreasing CVC below 10% among prevalent hemodialysis patients^2^. Although nationwide data in Turkey show that the > 66% goal has been achieved with a rate of 77.41%, the facilities included in this surveillance program had lower rates^1^. In our study, rate of the patients receiving HD via CVC was 42.2% and for these patients, the risk of bacteremia was 17.6 times higher than the patients with AV fistula. NHSN Dialysis Safety Network reported that the bloodstream infection (BSI) rate ratio between CVC and AV fistula was 8.2^3^. In our study population, proportion of patients receiving HD via catheter was higher than the rate reported in the national registry report. Since centers involved in the study are all tertiary hospitals’ HD units, patients’ characteristics can be different from the general population. Proportion of patients with tunneled catheter was high due to the centers included in the study being referral centers that predominantly give healthcare to complicated patients and existence of transient dialysis patients. Since the main goal of the study is not to compare infection rates across catheter types, baseline patient characteristics are different across these groups. These can explain lower infection rates obtained in non-tunneled CVC patients.

In 1999, CDC developed the Dialysis Surveillance Network for monitoring hemodialysis related infections. Definitions updated over the years and a few reports regarding the data have been published. In the recent years, several countries started to implement this surveillance system. Surveillance plays critical role for improving health care quality and safety of HD patients and can guide infection control programs, find the gaps where improvements might be needed. Moreover, this approach can raise the awareness of the healthcare workers regarding infection prevention. Gork et al.^4^ reported a significant decrease in dialysis related infections in a 9 years lasting study period. Their study consisted of both surveillance and intervention which includes checklists, ready kit for the care of vascular access, education, and an infection prevention team. They achieved a significant trend of decrease in access-related infection rates. Similarly, Yi et al.^5^ reported that participating a BSI prevention collaboration improves access related BSI rates in dialysis facilities. Integration a HD specific surveillance system in HD units can cause considerable decrease in HD related infections as well as antimicrobial consumption^6^. NHSN reported a significant decrease in BSI and access related infection rates in 2014^3^. However, rates of intravenous antimicrobial start were similar with previous reports. This result highlights the need for different efforts for achieving similar lower rates for antimicrobial consumption and lowering rates of colonization and infection of HD patients with multidrug resistant organisms. Tracking antimicrobial use and antimicrobial resistance of organisms in HD patients is essential for programs to prevent antimicrobial resistance.

As in surveillance of hospital-acquired infections, standard definitions and calculations are important in terms of monitoring and comparing units' own rates. NHSN did not report DE rates since 2014. Our DE rates for all types are lower than the rates reported by the NHSN in 2014 and 2011^3,7^. There are several reasons for lower DE rates. First, all centers participating in the study are at a tertiary care university hospital with high workload and they are all strictly controlled by the government. Benchmarking of data from a dialysis unit at a tertiary care university hospital with data from US outpatient dialysis units is not optimal, since there can be many differences in patient population, staff education and facility of the unit. On the other hand, NHSN reports significant differences in DE rates among facilities in US. Second, since this is the first study for surveillance of DE infections in our country, healthcare personnel are not familiar with this type of a data gathering system and this might have caused some underreporting. Even NHSN which implemented the system many years ago, discuss the quality of the data, problems understanding the system, and underreporting issue^8^. However, in our opinion implementation of an official nationwide surveillance system would have a significantly positive effect on data quality. Third, in NHSN report 2014, 6005 outpatient HD facilities reported data. Both the number of patients and the number of centers are very high when compared to our study. Therefore, it is not appropriate to compare these DE rates. There have been many differences among DE rates reported from different countries. Quebec Public Health Expertise and Reference Centre reported that their vascular access related BSI rate was 0.22 cases per 100 patient-periods^9^. They report that in 2016–2017, incidence rates for tunneled and non-tunneled catheters have significantly decreased compared to rates for 2012–2016 while rates for AV fistulas and grafts have remained stable. In a surveillance study from Kuwait reported rates of hospitalization, IV antimicrobial start, and positive blood culture were 4.3, 9.0 and 1.1 per 100 patient-months respectively^10^. In an Irish study from two HD units for a period of 6 weeks, rate of hospitalizations, IV antimicrobial starts, and positive blood cultures were 13, 8.52 and 3.14 per 100 patient months, respectively^11^. China reported 33 outpatient HD centers’ surveillance of dialysis events data in 2017^12^. Overall DE rate was 1.47 per 100 patients-months.

In contrast to published surveillance studies, we also searched vaccination rates for seasonal influenza and pneumococcal vaccine which are recommended for HD patients. Unfortunately, 32.2% for seasonal influenza vaccination and 5.8% for pneumococcal vaccination rates are both very low. Bond et al.^13^ reported that vaccination against influenza and pneumococcal disease is associated with improved survival in dialysis patients. Infection prevention strategies must include topics regarding raising vaccination rates for HD patients.

Most reported microorganisms responsible for BSI were coagulase-negative staphylococcus and *S. aureus*. This data is similar with NHSN’s and other studies’ data^3,9,10,12^. Rate of methicillin resistance among *S. aureus* was 46% in NHSN’s 2014 report and 1% in our report. In Qebec report *S. aureus* accounts most of the cases resulting in death (44%), however oxacillin resistance was 10.7% among *S. aureus*^9^. China reported 17.86% methicillin resistance among *S. aureus* isolates^12^.

The CDC published key interventions for prevention of BSI in HD patients. According to these recommendations, education for vascular access site care for all HD patients is essential. One of the most crucial results of this study is the low infection rate among patients who received education about the care of their vascular access site. This result revealed the importance of engaging patients for prevention strategies. Data on the HD patients’ perspective on infection prevention is limited in the literature. In a survey study, CDC staff evaluated the HD patients’ view and role on infection prevention strategies^14^. Participating patients concluded that patients should take the responsibility for their vascular access site care and should be observant for infection prevention steps. Ball et al.^15^ reported that patient engagement in the infection control practices causes significant reductions in BSI rates. HD patients spend much of their time in healthcare facilities, and they can have a positive effect for both their and other patients’ safety regarding infections^16^. In our study population, 80% of the patients were educated. Next step for lowering the BSI rates among HD patients must be to increase this rate to 100%.

Making Dialysis Safer for Patients Coalition was founded by CDC and CDC Foundation in 2016^17,18^. The coalition consists of a wide range of healthcare organizations and stakeholders and aims to prevent bloodstream infections in HD patients and raise awareness on recommended infection prevention practices. “Core Interventions for Bloodstream Infection Prevention” compiled by the coalition includes evidence-based practices for CVC care as well as benchmarking data collected through NHSN related with infection rate measures, education of staff and patients, audit, and competency assessments. Several centers reported significant, rapid and sustained reductions in DE rates after participating the Collaborative^5,19,20^. After the early success of the Collaborative was shown, CDC compiled checklists regarding the Core interventions used by Collaborative participants^21^. These checklists focused on hand hygiene and glove use, catheter exit site care, catheter connection and disconnection, arteriovenous fistula and graft cannulation and decannulation, and routine dialysis station disinfection. The most important outcome of our study was preparation of similar checklists for the Dialysis Services Unit of the Turkish Ministry of Health. After evaluating our project, Ministry of Health General Directorate of Health Services officially distributed these checklists to all dialysis centers in Turkey. Moreover, they made it mandatory to complete checklists for each HD patient. Completing this checklist for every HD session of each patient has raised the awareness of healthcare workers, since the use of checklist is an application that has proven to be effective in reducing infection rates^22–24^. This was an unexpected result of utmost impact that we neither have intended nor foreseen at the beginning of the study.

This study has some limitations. First, like NHSN, we included data from all participating HD units regardless of the number of months reported. This can certainly lower the quality of the data. Second, all centers participating in the study are tertiary hospitals. Therefore, rates may not reflect the national data. Third, we conducted the study with a limited number of clinicians and research staff. High workload at these centers might have increased the probability of underreporting.

In conclusion, hemodialysis units are not covered in the National Nosocomial Infection Network run by the Turkish Ministry of Health General Directorate of Public Health. This first surveillance study revealed the baseline status of HD related infections in Turkey and showed that NHSN DE surveillance system can be easily implemented even in a high workload dialysis unit and be adopted as a nationwide DE surveillance program. Results have highlighted the importance of optimizing vascular access, appropriate care of catheters and the patient education for vascular access site care. Awareness of healthcare workers regarding infections in HD patients is one of the most important points of preventing, and this study provided a great contribution for raising awareness of healthcare workers in dialysis units. Revealing that DE rates are lower in patients who are educated about the care of their vascular access site will hopefully make healthcare workers more attentive. While there are caveats with international comparisons as discussed above, we have established a baseline that will facilitate us to demonstrate the effect of future infection prevention and control and antimicrobial stewardship strategies.

## Materials and method

A multicenter prospective surveillance study was performed to investigate the infection rates among HD patients. We implemented a central database for data collection with a web interface compatible with mobile devices called TR-HIES (www.tr-hies.org). CDC NHSN DE protocol was adopted for definitions and reporting^25^. All patients, including transient patients taking dialysis in the HD unit were included in the study. After the patient information form in the system was filled, patients were followed for DE. The network’s computer algorithm determined if case definitions for infection were met.

**Dialysis event**: Three DEs are defined:

1. **Use of intravenous antibiotic**: Antibiotics and antifungals initiated in the patient are reported independently of the cause and duration of treatment. Patient should be entered as intravenous antibiotic use in dialysis related events even if he or she has taken treatment for 1 day. Antivirals and oral antibiotics are not included.
2. **Positive blood culture:** All positive blood cultures of outpatients and all positive blood cultures that have been taken 1 day after the admission to the hospital of inpatients are recorded. According to CDC protocol, all positive blood cultures shall be reported even if it is thought not to be a true infection or unrelated to hemodialysis^25^.
3. **Local vascular access site infection:** All patients who have one or more symptoms of pus, redness or swelling at vascular access site are reported regardless of whether the patient received treatment for infection or not.

According to the CDC DE protocol, 21-day rule was applied to prevent two events that may be related to each other from being reported as different events. 21-day rule requires an event to be considered as an individual event if and only if at least 21 days have passed after a possible previous event (of the same patient).

### Data analysis

To calculate DE rate, the number of DE developed within that month and the total number of patients received HD during the first 2 working days of the same month are recorded. DE rates are calculated for each vascular access type and given as per 100 patient months. If the patient had more than one vascular access, only their vascular access type with the highest risk of infection was reported. Access types from lowest infection risk to highest are: fistulas, grafts, tunneled central lines, and non-tunneled central lines^25^.

The web-based data entry application developed for the study prohibited entry of events with missing data to ensure integrity of the event data. Furthermore, in the case of missing required (within a limited time period) follow-up data, the event was only used in analysis where only its occurrence is relevant.

### Additional data

In addition to the CDC protocol, some patient characteristics were also evaluated. The center coordinator was asked to record some characteristics such as age, gender, duration of dialysis, comorbid diseases, and vaccination status while recording each patient in the database.

This study was approved by the Ethics Committee of Yildirim Beyazit University. All methods were carried out in accordance with relevant guidelines and regulations. A waiver of informed consent from Yildirim Beyazit University ethics committee was obtained for the surveillance data.

### Statistical analysis

Results were analyzed with SAS JMP® statistical software package (version 11, available at: https://www.jmp.com/). Bivariate correlations among all variables were calculated in the multivariate analysis. Comparisons between groups for continuous variables were performed with Student’s t-test if they were distributed normally and with Kruskal–Wallis test if they were not distributed normally. Nominal variables were compared with Pearson χ^2^ and Fisher’s Exact test. A *p* value below 0.05 was considered statistically significant.
